# Endovascular treatment of a teenager with nutcracker syndrome: a case report

**DOI:** 10.1590/1677-5449.180126

**Published:** 2020-05-08

**Authors:** Sergio Quilici Belczak, Léa Luz, Luciana Barbosa Paglia, Gabriela Prata Ramos Barbosa, Alex Mendes Leonel Freire, Matheus Amparado Miziara, Paulo Eduardo Baldini Lucena, Douglas Yuji Saito

**Affiliations:** 1 Centro Universitário São Camilo, São Paulo, SP, Brasil.; 2 Instituto de Aprimoramento e Pesquisa em Angiorradiologia e Cirurgia Endovascular – IAPACE, São Paulo, SP, Brasil.

**Keywords:** renal nutcracker syndrome, renal vein, hematuria

## Abstract

The nutcracker syndrome is caused by compression of the left renal vein by the superior mesenteric artery and aorta and is associated with characteristic symptoms, such as lower abdominal pain, varicocele, and hematuria. Diagnosis is often difficult and, therefore, is often delayed. Invasive treatment is controversial, particularly in pediatric patients. However, it is indicated in cases of gross hematuria associated with anemia, renal function impairment, severe pelvic pain, or ineffective conservative treatment. We report the case of a 12-year-old boy presenting with severe hematuria for 12 hours, with no abnormal findings at a first evaluation, who progressed with severe anemia and urinary retention. Further investigation provided images suggestive of nutcracker syndrome, and endovascular stenting (smart control stent) followed by balloon dilatation was the treatment of choice. Hematuria ceased after the procedure, and the patient is still asymptomatic at 5-year follow-up.

## INTRODUCTION

The nutcracker syndrome is a difficult-to-diagnose condition that is often identified late^1^ and is characterized by a group of clinical manifestations caused by compression of the left renal vein (LRV) between the superior mesenteric artery (SMA), anteriorly, and the aorta (AA), posteriorly.^2^ The normal angle between the SMA and the aorta is 90°, but the LRV is compressed when this angle is acute, giving rise to the anterior nutcracker syndrome, which accounts for the majority of cases. There are also descriptions in the literature of posterior nutcracker syndrome, which occurs when the course of the renal vein is retroaortic or circumferential to the aorta, with compression occurring between the aorta and the vertebral body.^3^

The syndrome is most common among women aged from 20 to 40 years. The most frequent symptom is hematuria, followed by abdominal and flank pains, which can spread to thighs and buttocks. Pain may worsen in sitting and standing positions.^1^

Imaging exams are essential for diagnosis, and the most widely-used methods for this purpose are renal Doppler ultrasonography and computed tomography angiography.^4^ Treatment for nutcracker syndrome is debatable, with clinical and surgical options, depending on the severity of the symptoms.^5^ The emergence of endovascular surgery has enabled less invasive interventions with lower morbidity, and stenting is often used in these cases.^2^

This report presents the case of a 12-year-old patient with a diagnosis of nutcracker syndrome who was treated successfully with stenting of the LRV.

## CASE DESCRIPTION

A 12-year-old male patient was admitted via the urology service after presenting with gross hematuria lasting 12 hours. He had no other complaints and mentioned nothing of note in relation to personal or family history. Computed tomography was ordered, but showed nothing, according to the report. A blood test conducted at admission showed serum hemoglobin (Hb) of 11.7 mg/dL.

Over 24 hours, the patient developed intense anemia, reaching Hb of 7.4 mg/dL, and urinary retention. Urethral catheterization was performed to relieve the bladder, eliminating countless clots, and ultrasonography revealed a large clot in the bladder interior.

A transfusion was then performed with two packed red blood cell units, and cystoscopy was conducted with bladder lavage and removal of clots. Active blood flow was observed from the left ureter into the interior of the bladder and so the urology team inserted a JJ catheter.

**Figure 1:**
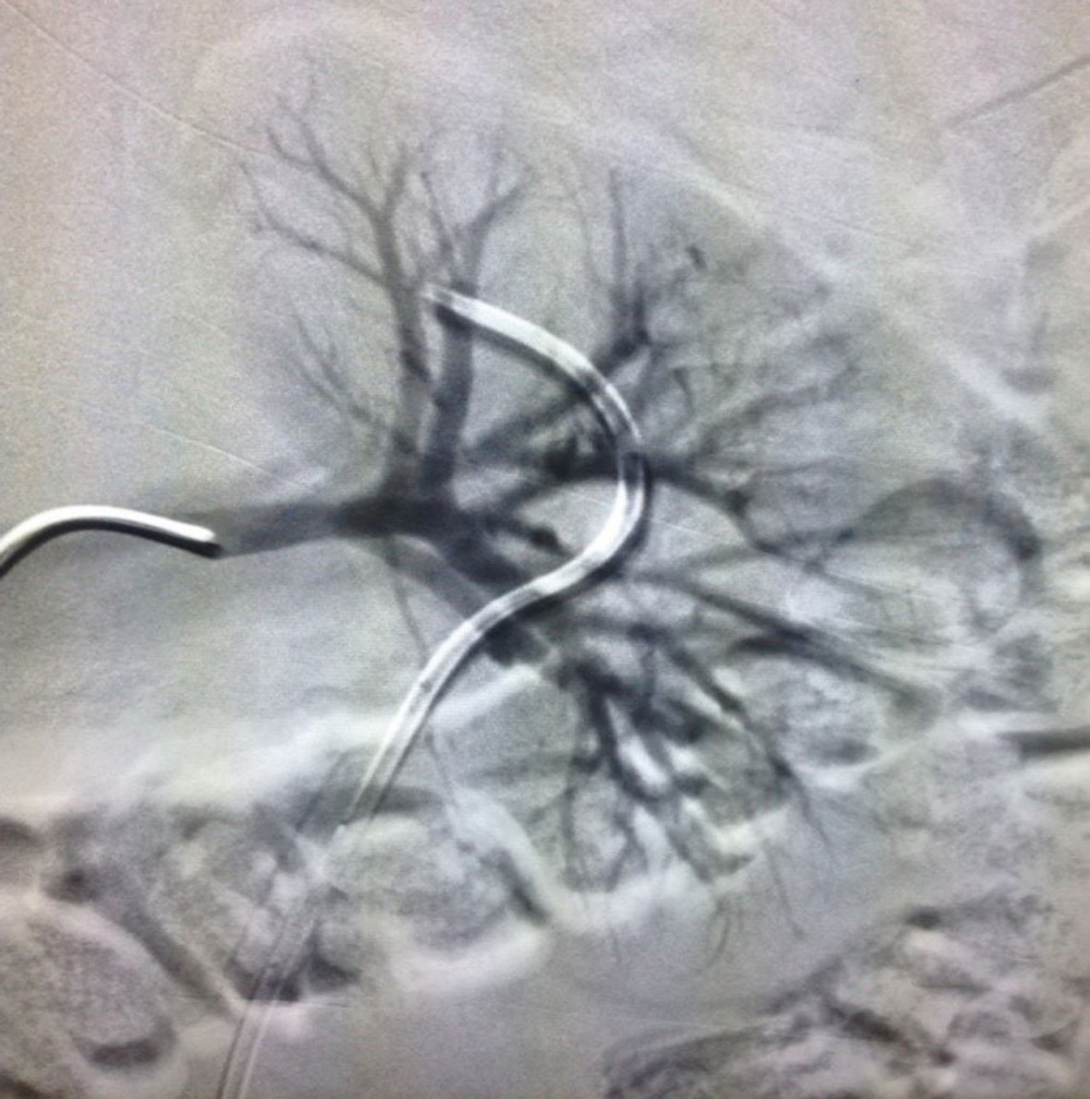
Arteriography of the left renal artery.

**Figure 2:**
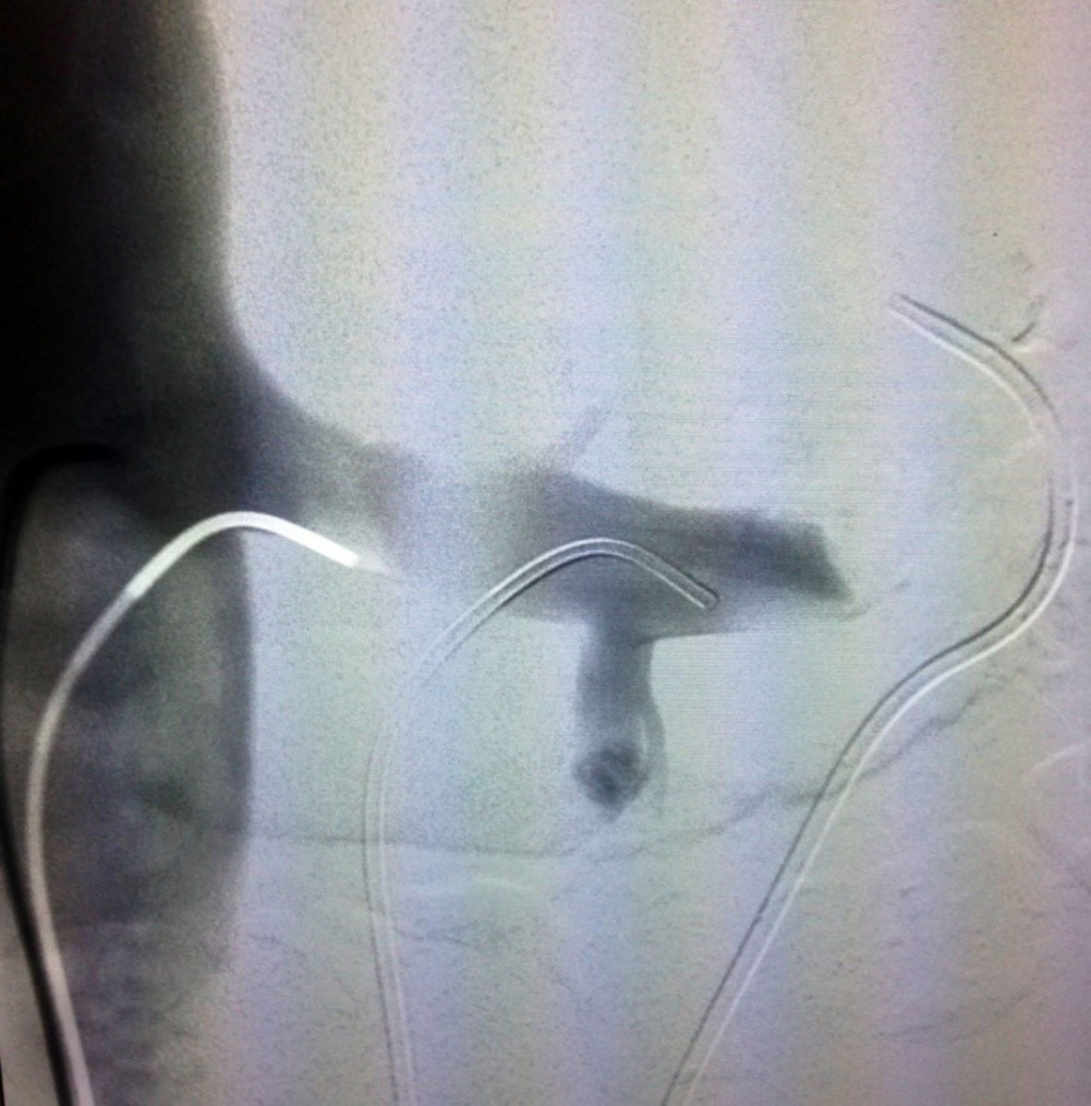
Phlebography of the left renal vein, showing reduced outflow and stenosis.

**Figures 3:**
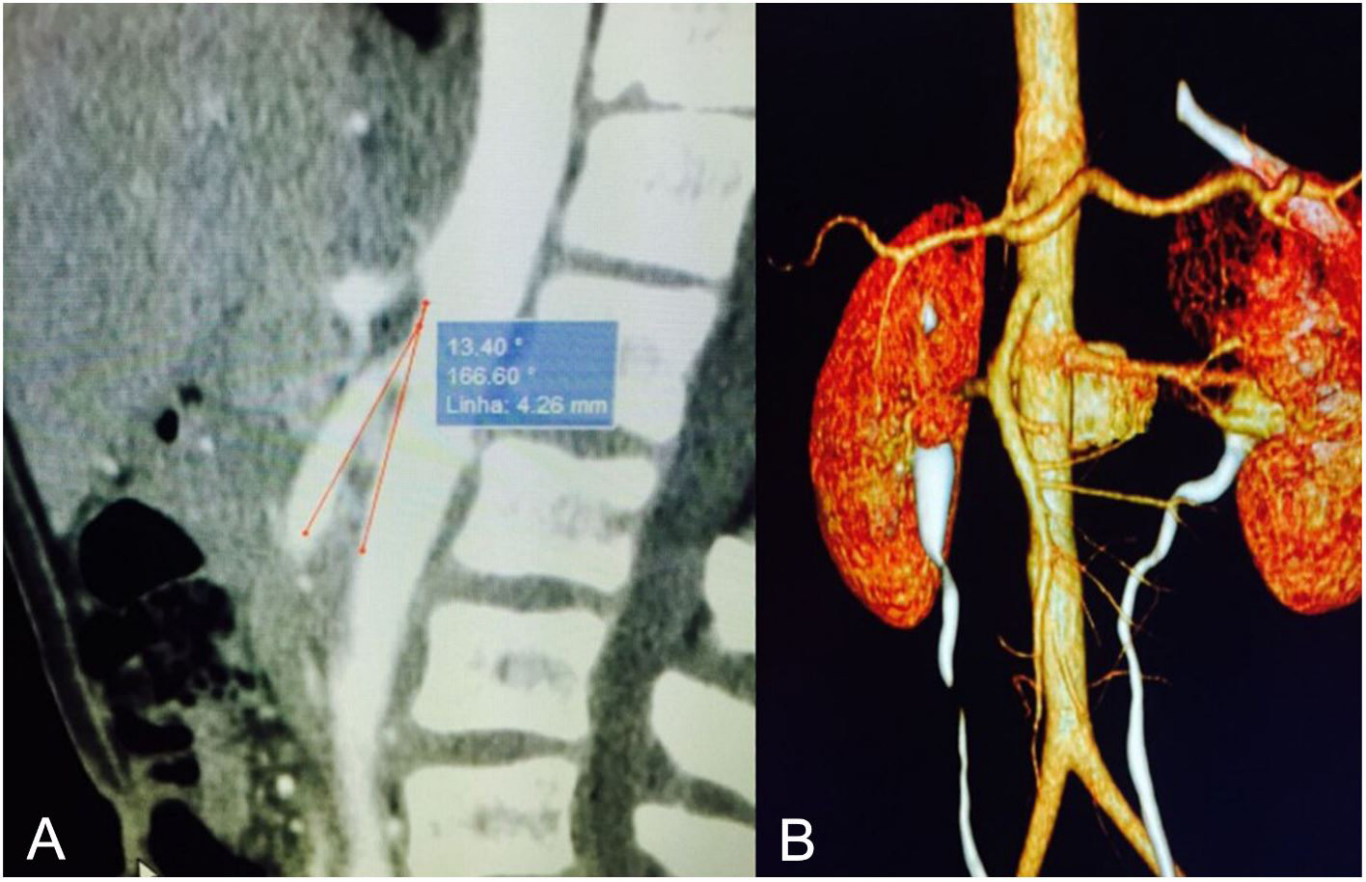
The initial computed tomography (A) and three-dimensional reconstruction (B) re-assessed, showing compression of the left renal vein.

Arteriography was ordered and ruled out arteriovenous malformations and renal arteriovenous fistulas (Figure 1). However, it showed very slow renal drainage, considerable stenosis of the LRV, and images revealing compression of the SMA, suggestive of nutcracker syndrome (Figure 2). Reassessment of the initial computed tomography revealed compression of the LRV by the SMA, with an acute exit angle, of approximately 13.4º (Figure 3).

**Figure 4:**
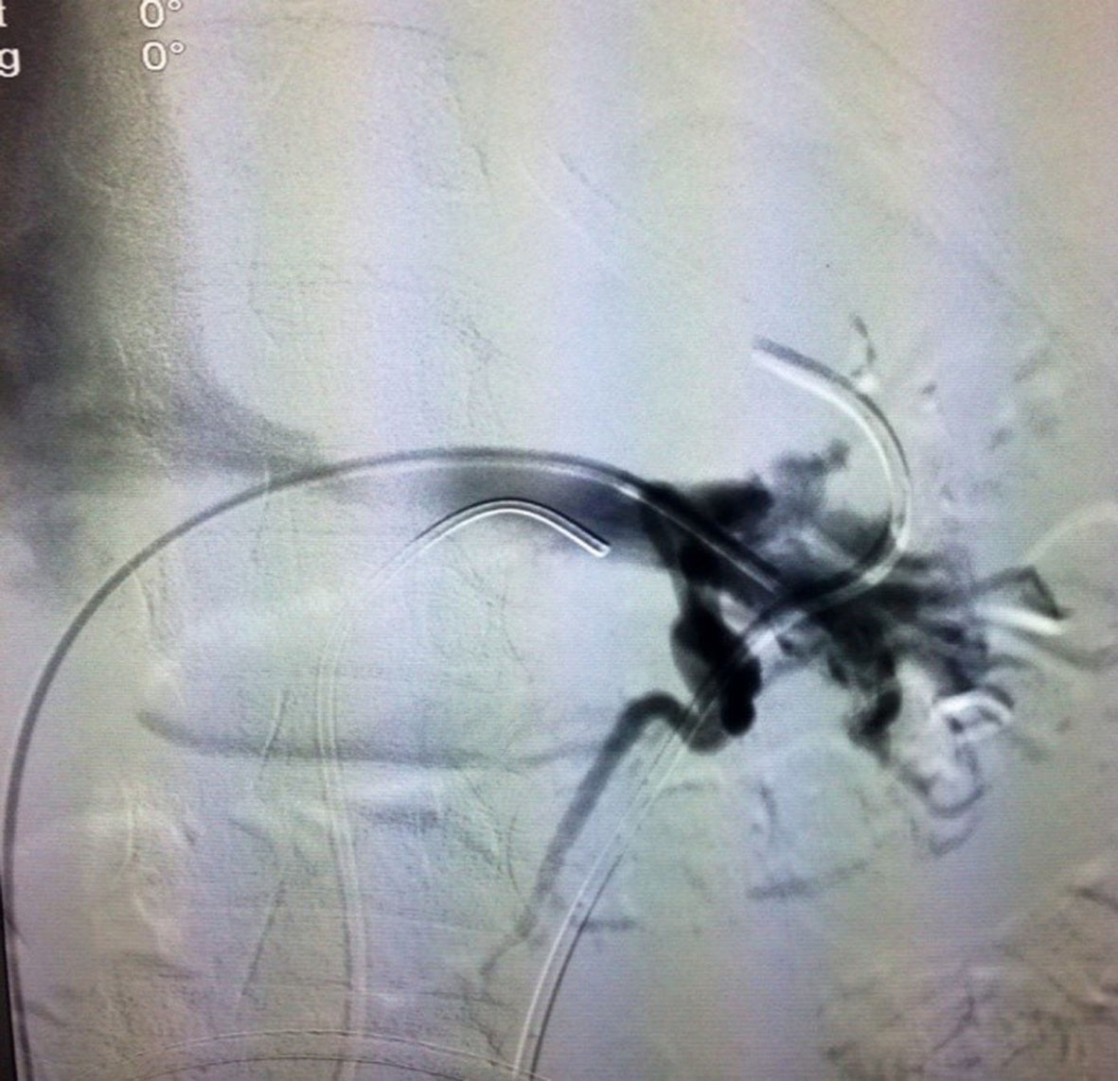
Phlebography after 10 × 40 mm balloon angioplasty, showing that stenosis remains.

**Figure 5:**
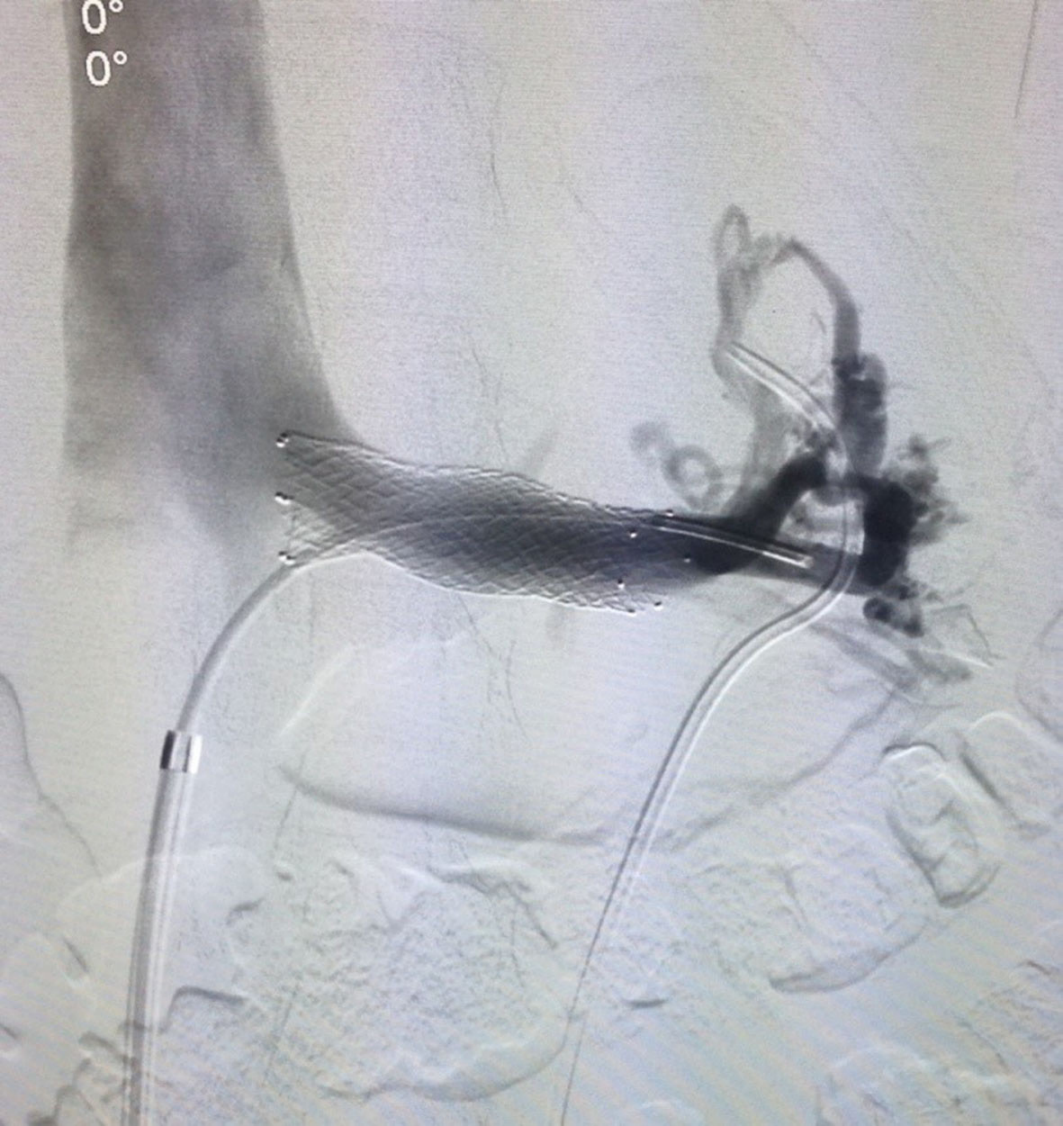
Deployment of the smart control stent followed by 10 × 40 mm balloon angioplasty.

Repair was attempted by angioplasty with a 10 × 40 mm balloon, but there was considerable recoil, and stenosis remained (Figure 4). The case was discussed with the urology team, assessing the possibility of nephrectomy. Instead, the decision was taken to deploy a 12 × 40 mm smart control stent, followed by ballooning once more, with a 10 × 40 mm balloon (Figure 5).

**Figure 6:**
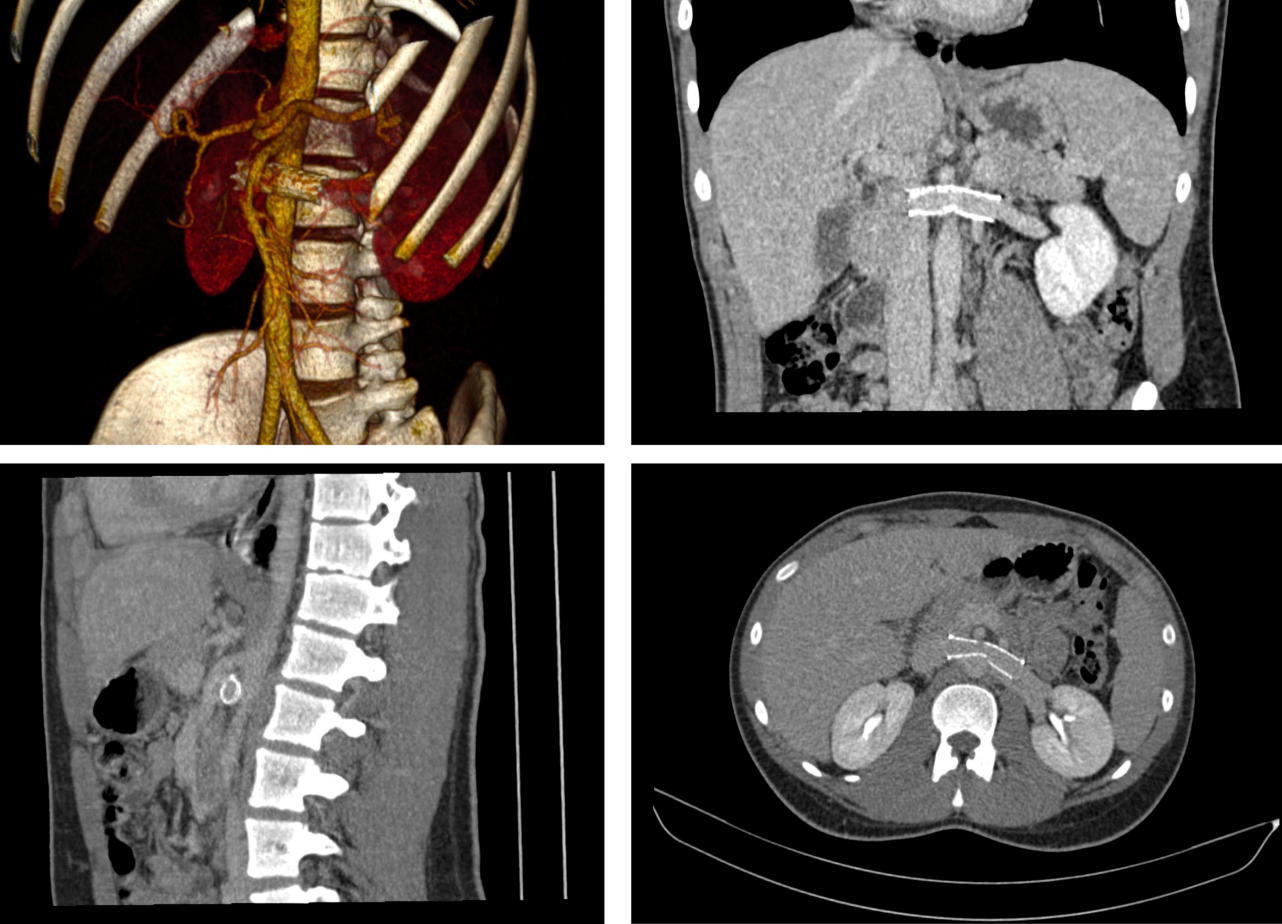
Control angiotomography images, 5 years after the procedure.

The patient’s hematuria ceased completely in 6 hours, even though he was on acetylsalicylic acid (ASA) and clopidogrel. The patient has been asymptomatic for 5 years, with control angiotomography showing the stent patent and no compressions (Figure 6).

## DISCUSSION

Initially described in 1950, the nutcracker syndrome typically consists of compression of the LRV between the aorta and the SMA.^2^^,^^3^ It is believed that the syndrome is associated with a lack of retroperitoneal fat, which reduces the exit angle of the SMA, or nephroptosis, causing elongation of the LRV.^2^ There is also another variant of the syndrome, in which the LRV is compressed between the aorta and the spine in the presence of an anatomic variant with a retroaortic LRV position. Compression of the LRV, and its consequent stenosis, lead to varying degrees of flow reduction, causing venous hypertension. This may be asymptomatic or may cause a range of signs and symptoms, such as hematuria (rupture of the thin walls of vessels adjacent to the collector system manifests clinically as microscopic or macroscopic hematuria), lumbar pain, left flank pain, spreading to the posterolateral thigh and the buttocks, orthostatic proteinuria, and left-side varicocele.^2^^,^^3^ The most common symptom of the syndrome is hematuria, varying from microscopic to bleeding linked to anemia, as in the present report, in which the patient’s Hb dropped from 11.7 to 7.4 mg/dL over the 24 hours after admission.

Compression of the LRV can also be caused by pancreatic cancer, periaortic lymphadenopathy, retroperitoneal cancer, or presence of localized fibrotic tissue between the aorta and the SMA. Prevalence is higher among females and it can have onset in childhood or adulthood, but frequency is highest in from the 2^nd^ to 3^rd^ decades of life.^3^

Symptoms can be aggravated by physical activity and other clinical conditions are common, primarily kidney stones. Nutcracker syndrome is difficult to diagnose, and it is necessary to first investigate and rule out other more common and/or serious causes of the symptoms, particularly of hematuria.^2^^,^^3^ Presence of hematuria from the orifice of the left ureter in the absence of any other detectable pathology should arouse suspicion. Cystoscopy can therefore assist in diagnosis, but it may fail to detect intermittent hematuria. The cystoscopy conducted for our patient showed blood actively exiting into the interior of the bladder. Laboratory urine cytochemistry analysis tests can only identify hematuria and proteinuria.

Arteriography was ordered to test for arteriovenous malformations or renal arteriovenous fistula, which could have been the cause of gross hematuria, and ruled out these conditions. Clinical and biochemical characteristics may not be evident and detection of hypertension in the left renal vein by radiological procedures is subject to operator error, to the extent that the syndrome may be more common than is described in the literature.^6^ In children, Doppler ultrasound and magnetic resonance angiography are the first choice tests because they are innocuous and generally lead to diagnosis.^7^ Phlebography with intravascular ultrasonography has also proved to be an important tool for diagnostic confirmation and for intraoperative assessment of the result of stenting and of relief of extrinsic compression.^8^

Prognosis is variable with nutcracker syndrome and is dictated by the magnitude of compression of the renal vein. In some cases, development of collateral venous circulation can trigger regression of the symptomatology.^7^

Since hematuria will resolve in 75% of cases, treatment should be conservative for at least 2 years in patients under the age of 18 years. However, in cases with severe symptoms, interventional treatment should be provided and this encompasses a range of surgical options, including nephropexy and renal vein bypass, transposition of the left renal vein, with or without dacron reinforcement, transposition of the superior mesenteric artery, kidney autotransplantation and renal vein bypass to the vena cava, through gonadocaval bypass, or even nephrectomy.^3^

Placement of external or intravascular stents has been described with good results. The current tendency to favor minimally invasive surgery suggests that intravascular stenting could be beneficial for patients, but care should be taken to avoid intimal hyperplasia and later occlusion of the stent. Therefore, it is recommended that platelet antiaggregants or anticoagulants should be used for at least 2 to 3 months, while waiting for full neoendothelialization of the site. There are few publications describing implantation of venous stents in young patients and there is no consensus on this application. While there are reports of promising results so far, there is little evidence on the long-term patency of these stents in these patients.^9^^,^^10^ Since adolescent and pediatric patients will continue to grow, growth of the blood vessel could lead to local stenosis and this factor should be taken into account whenever endovascular treatment is indicated. Similarly, there are several reports of other complications, especially migration of stents.^11^ There is recent evidence of treatment of patients with nutcracker syndrome in retroaortic renal veins, with results similar to those observed in patients who have undergone endovascular treatment for anterior nutcracker syndrome.^12^

The results in the case presented here were satisfactory. After deployment of a 12 × 40 mm smart control stent, followed by 10 × 40 mm balloon angioplasty, the patient’s hematuria ceased completely in 6 hours, even though he was on platelet antiaggregants and anticoagulants. The patient has remained asymptomatic for 5 years.
