# Supplementary material for: Endovascular treatment of a teenager with nutcracker syndrome: a case report
Source: J Vasc Bras. 2020 May 8;19:e20180126. doi: 10.1590/1677-5449.180126 (PMC8202204; doi:10.1590/1677-5449.180126)
Supplement: Translation

# Translation: Tratamento endovascular da síndrome do quebra-nozes em adolescente: relato de caso

**Authors:** Sergio Quilici Belczak, Léa Luz, Luciana Barbosa Paglia, Gabriela Prata Ramos Barbosa, Alex Mendes Leonel Freire, Matheus Amparado Miziara, Paulo Eduardo Baldini Lucena, Douglas Yuji Saito

**Affiliations:** 1 Centro Universitário São Camilo, São Paulo, SP, Brasil.; 2 Instituto de Aprimoramento e Pesquisa em Angiorradiologia e Cirurgia Endovascular – IAPACE, São Paulo, SP, Brasil.

**Keywords:** síndrome do quebra-nozes, veias renais, hematúria

## Abstract

A síndrome do quebra-nozes (ou síndrome de *nutcracker*) é causada pela compressão da veia renal esquerda pela artéria mesentérica superior e aorta, e está associada a uma sintomatologia característica, como dor no baixo ventre, varicocele e hematúria. O diagnóstico é frequentemente difícil e, portanto, demorado. O tratamento invasivo é controverso, especialmente nos pacientes pediátricos; no entanto, em casos de hematúria severa associada a anemia, insuficiência renal funcional, severa dor pélvica ou ineficácia de tratamento conservador, ele é indicado. É relatado o caso de uma criança do sexo masculino, 12 anos, com quadro de hematúria maciça por 12 horas, sem evidências de alterações à investigação inicial, que evoluiu com anemia intensa e retenção urinária. Investigações futuras evidenciaram imagens sugestivas da síndrome de *nutcracker* e foi optado pelo tratamento endovascular por implante de stent *smart control* seguido de balonamento. Paciente cessou a hematúria após o procedimento e permanece assintomático há 5 anos.

## INTRODUÇÃO

A síndrome do quebra-nozes, também conhecida como *nutcracker*, é uma condição de diagnóstico difícil e frequentemente tardio^1^, sendo caracterizada por um conjunto de manifestações clínicas que ocorrem devido à compressão da veia renal esquerda (VRE) pela artéria mesentérica superior (AMS), anteriormente, e pela artéria aorta (AA), posteriormente^2^. O ângulo normal entre a AMS e a AA é de 90º; porém, quando esse ângulo é agudo, ocorre a compressão da VRE, originando o “quebra-nozes” anterior, correspondente à maioria dos casos. Na literatura, também é descrito o “quebra-nozes” posterior, que ocorre quando a veia renal se encontra retroaórtica ou circunferencial à aorta, e a compressão se dá pela aorta e pelo corpo vertebral^3^.

Essa síndrome é mais comum em mulheres entre 20 e 40 anos. O sintoma mais frequente é a hematúria, seguido de dor abdominal e nos flancos, com possível irradiação para coxas e nádegas. A dor pode apresentar piora nas posições sentada e ortostática^1^.

Exames de imagem são essenciais no diagnóstico, e os mais utilizados são a ultrassonografia com Doppler renal e a angiotomografia computadorizada^4^. O tratamento da síndrome do quebra-nozes é discutível, podendo ser clínico ou cirúrgico, dependendo da gravidade dos sintomas apresentados^5^. Com o surgimento da cirurgia endovascular, o procedimento intervencionista passou a ser menos invasivo e de menor morbidade, sendo a colocação de stents muito utilizada nesses casos^2^.

Este relato tem como objetivo apresentar o caso de um paciente de 12 anos com diagnóstico de síndrome do quebra-nozes, tratado com sucesso por implante de stent na VRE.

## DESCRIÇÃO DO CASO

Paciente do sexo masculino, 12 anos, deu entrada pelo serviço de urologia com quadro de hematúria maciça havia 12 horas. Negava outros tipos de queixas e não relatava nada digno de nota em antecedentes pessoais e familiares. Foi solicitada tomografia computadorizada, que não evidenciou alterações segundo laudo, e também um hemograma com nível sérico de hemoglobina (Hb) de entrada de 11,7 mg/dL.

Em 24 horas, o paciente evoluiu com anemia intensa, apresentando Hb de 7,4 mg/dL e quadro de retenção urinária. Assim, foi realizada sondagem uretral de alívio com eliminação de inúmeros coágulos, além de uma ultrassonografia que evidenciou um grande coágulo no interior da bexiga.

Em seguida, realizou-se transfusão com dois concentrados de hemácias e cistoscopia com lavagem vesical e retirada de coágulos. Evidenciou-se saída ativa de sangue pelo ureter esquerdo no interior da bexiga e, então, foi passado cateter duplo J pela equipe de urologia.

Foi solicitada arteriografia, que descartou malformações arteriovenosas e fístula arteriovenosa renal (Figura 1). Porém, evidenciou-se escoamento renal bem lentificado, estenose importante de VRE e imagem de compressão da AMS, sugestiva de síndrome de *nutcracker* (Figura 2). Ao se reavaliar a tomografia computadorizada inicial, observou-se, então, compressão da VRE pela AMS com ângulo de emergência agudo de aproximadamente 13,4º (Figura 3).

**Figura 1:** Arteriografia de artéria renal esquerda.

**Figura 2:** Flebografia de veia renal esquerda evidenciando diminuição do escoamento e estenose.

**Figuras 3:** Tomografia computadorizada inicial (A) e reconstrução tridimensional (B) reavaliadas demonstrando compressão da veia renal esquerda.

Para correção, foi realizada angioplastia com balão 10 × 40 mm, porém com *recoil* importante e manutenção da estenose (Figura 4). O caso foi levado para discussão com a equipe de urologia, avaliando-se a possibilidade de nefrectomia. Optou-se pelo implante de stents *smart control* 12 × 40 mm, seguido de novo balonamento com balão 10 × 40 mm (Figura 5).

**Figura 4:** Flebografia após angioplastia com balão 10 × 40 mm evidenciando manutenção da estenose.

**Figura 5:** Implante do stent *smart control* seguido de angioplastia com balão 10 × 40 mm.

A hematúria do paciente cessou completamente em 6 horas, mesmo com o uso de ácido acetilsalicílico (AAS) e clopidogrel. Há 5 anos, o paciente permanece assintomático e com angiotomografia de controle evidenciando perviedade do stent e ausência de compressões (Figura 6).

**Figura 6:** Imagens de Angiotomografia de controle de 5 anos após o procedimento.

## DISCUSSÃO

Inicialmente descrita em 1950, a síndrome do quebra-nozes (*nutcracker*) consiste na típica compressão da VRE pela AA e pela AMS^2^^,^^3^. Acredita-se que a escassez de gordura retroperitoneal, levando a uma diminuição do ângulo de emergência da AMS, ou a nefroptose, levando a um alongamento da VRE, estejam associadas à síndrome^2^. Há também um segundo tipo da Síndrome causado pela compressão da VRE entre a aorta e a coluna quando esta apresenta uma variação anatômica configurando uma posição retroaórtica. A compressão da VRE e consequentemente sua estenose, leva em diferentes graus, a redução do seu fluxo, provocando hipertensão venosa. Esta pode ser assintomática ou gerar diversos sinais e sintomas, como a hematúria (a ruptura das finas paredes dos vasos adjacentes ao sistema coletor traduz-se clinicamente em micro-hematúria ou hematúria macroscópica), dor lombar, dor em flanco esquerdo com irradiação para a região posterolateral da coxa e para as nádegas, proteinúria ortostática e varicocele esquerda^2^^,^^3^. O sintoma mais comum da síndrome é a hematúria, que varia de micro até sangramentos associados à anemia, como no presente relato, no qual a Hb do paciente evoluiu de 11,7 para 7,4 mg/dL após 24 horas de internação.

A compressão da VRE pode ocorrer devido a neoplasia pancreática, linfadenopatia periaórtica, neoplasias retroperitoneais ou presença de tecido fibrótico local entre a AA e a AMS. Sua prevalência é superior no sexo feminino, podendo aparecer na infância ou na idade adulta, porém mais frequentemente entre a 2ª e a 3ª décadas de vida^3^.

Os sintomas podem ser agravados com atividade física e são comuns a outras situações clínicas, principalmente a nefrolitíase. O diagnóstico da síndrome do quebra-nozes é difícil, exigindo a investigação prévia de outras causas mais frequentes e/ou mais graves desses sintomas (principalmente a hematúria)^2^^,^^3^. A presença de hematúria no orifício ureteral esquerdo, na ausência de qualquer outra patologia detectável, deve levantar a suspeita. A cistoscopia, então, pode auxiliar o diagnóstico, mas pode não revelar hematúria intermitente durante o exame. Na cistoscopia realizada no nosso paciente, observou-se a saída de sangue ativamente no interior da bexiga. Laboratorialmente, a hematúria e a proteinúria são os únicos elementos identificáveis na análise citoquímica da urina.

Para descartar malformações arteriovenosas ou fístula arteriovenosa renal, que poderiam justificar a hematúria maciça, foi solicitada uma arteriografia, que descartou as lesões. As características clínicas e bioquímicas podem não ser evidentes, além de a detecção da hipertensão da veia renal esquerda por procedimentos radiológicos estar sujeita à falha do operador, de forma que a síndrome possa ser mais comum do que se encontra na literatura^6^. Em crianças, a ecografia com Doppler e a angiorressonância são de primeira escolha pela inocuidade e por geralmente serem esclarecedoras do diagnóstico^7^. A realização de flebografia com a ulilização de ultrassonografia intravascular também tem se mostrado ferramenta importante para confirmação do diagnóstico e avaliação intraoperatória do resultado do implante do stent e alívio da compressão extrínseca^8^.

O prognóstico da Síndrome de Nutcracker é variável e ditado pela magnitude da compressão da veia renal. Em alguns casos, o desenvolvimento de circulação venosa colateral desencadeia uma regressão da sintomatologia^7^.

Uma vez que em 75% dos casos a hematúria se resolve, o tratamento deve ser conservador durante pelo menos 2 anos em pacientes com idade abaixo de 18 anos. No entanto, em casos com sintomas graves, o tratamento intervencionista deve ser realizado e compreende várias opções de cirurgias, incluindo desde nefropexia e *bypass* da veia renal, passando por transposição da veia renal esquerda com ou sem reforço de dacron, transposição da artéria mesentérica superior, autotransplante renal e derivação da veia renal para a veia cava, até *bypass* gonadocaval ou mesmo nefrectomia^3^.

Foi descrita, com bom resultado, a colocação de stent externo ou intravascular. Atualmente, a tendência de cirurgia minimamente invasiva sugere que o *stent* intravascular pode ser benéfico para o paciente, mas deve-se tomar cuidado com hiperplasia intimal e posterior oclusão do *stent*. Para tanto, aconselha-se o uso de antiagregantes plaquetários ou anticoagulantes por pelo menos 2 a 3 meses, a fim de aguardar a efetiva neoendotelização local. Há poucas publicações em relação ao implante de stents venosos em pacientes jovens, e falta de consenso nessa indicação. Mas até então, há relatos de resultados promissores. Porém não há muitas evidências sobre a perviedade destes *stents* a longo prazo nestes indivíduos^9^^,^^10^. Uma vez que pacientes adolescentes e pediátricos ainda vão crescer, o crescimento do vaso sanguíneo pode acarretar uma estenose no local, esse fator deve ser considerado se terapia endovascular foi indicada. Da mesma forma, há diversos relatos de outras complicações, especialmente migração de *stents* foram descritos^11^. Há também evidências atuais do tratamento inclusive de pacientes com quebra-nozes em veia renal retroaórtica, com resultados semelhantes àqueles obtidos em pacientes submetidos ao tratamento endovascular em quebra-nozes anterior^12^.

No presente caso apresentado, o resultado foi satisfatório. Após implante de *stent smart control* 12 × 40 mm, seguido de angioplastia com balão 10 × 40 mm, a hematúria do paciente cessou completamente em 6 horas, mesmo com uso de antiagregantes plaquetários e anticoagulantes. Há 5 anos, o paciente permanece assintomático.
